# The effect of dietary zinc - and polyphenols intake on DMBA-induced mammary tumorigenesis in rats

**DOI:** 10.1186/1423-0127-19-43

**Published:** 2012-04-16

**Authors:** Barbara Bobrowska-Korczak, Dorota Skrajnowska, Andrzej Tokarz

**Affiliations:** 1 Department of Bromatology, Faculty of Pharmacy, Medical University of Warsaw, Banacha 1, Warsaw 02-097, Poland

**Keywords:** Breast cancer, Tissue minerals, Zinc, Polyphenols

## Abstract

**Background:**

The aim of the study was to investigate the effect of dietary supplementation with zinc and polyphenol compounds, i.e. resveratrol and genistein, on the effectiveness of chemically induced mammary cancer and the changes in the content of selected elements (Zn, Cu, Mg, Fe, Ca) in tumors as compared with normal tissue of the mammary gland.

**Methods:**

Female Sprague-Dawley rats were divided into study groups which, apart from the standard diet and DMBA (7,12-dimethyl-1,2- benz[a]anthracene), were treated with zinc ions (Zn) or zinc ions + resveratrol (Zn + resveratrol) or zinc ions + genistein (Zn + genistein) via gavage for a period from 40 days until 20 weeks of age. The ICP-OES (inductively coupled plasma optical emission spectrometry) technique was used to analyze the following elements: magnesium, iron, zinc and calcium. Copper content in samples was estimated in an atomic absorption spectrophotometer.

**Results:**

Regardless of the diet (standard; Zn; Zn + resveratrol; Zn + genistein), DMBA-induced breast carcinogenesis was not inhibited. On the contrary, in the Zn + resveratrol supplemented group, tumorigenesis developed at a considerably faster rate. On the basis of quantitative analysis of selected elements we found - irrespectively of the diet applied - great accumulation of copper and iron, which are strongly prooxidative, with a simultaneous considerable decrease of the magnesium content in DMBA-induced mammary tumors. The combination of zinc supplementation with resveratrol resulted in particularly large differences in the amount of the investigated elements in tumors as compared with their content in normal tissue.

**Conclusions:**

Diet supplementation with zinc and polyphenol compounds, i.e. resveratrol and genistein had no effect on the decreased copper level in tumor tissue and inhibited mammary carcinogenesis in the rat. Irrespectively of the applied diet, the development of the neoplastic process in rats resulted in changes of the iron and magnesium content in the cancerous tissue in comparison with the healthy mammary tissue. The application of combined diet supplementation with zinc ions and resveratrol considerably promoted the rate of carcinogenesis and increased the number of DMBA-induced mammary tumors.

## Background

The disturbances in minerals homeostasis may induce biochemical changes characteristic of many diseases, including neoplastic diseases [1]. For instance, dietary insufficiencies in **selenium, zinc and magnesium **elements, and excess intake of **copper **and **iron **may increase the cancer risk [2-5]. It was **uncovered **in many investigations that in mammary malignant tumors an increased copper content is found in comparison with normal tissue and that ceruloplasmin level significantly increases in neoplastic diseases [6-8]. High copper concentration results in increased oxidative stress in the cells, and thus to modification of many cell structures [6]. Copper has high affinity to DNA, it has the strongest ability to bind to DNA of all other divalent cations and it can impair DNA structure via oxidation. These impairments together with inadequate rate of the biological system's ability to repair the resulting damage and with simultaneous permanent exposure to the reactive oxygen species may result in the onset of the process of carcinogenesis. In addition, copper plays a considerable role in the process of angiogenesis of malignant cells, as it is indispensable for activation of endothelial cells. Many compounds characterized by various mechanisms of inhibiting the development of blood vessels have already been introduced into clinical trials. Copper has become one of therapeutic targets in antiangiogenic anticancer treatment.

In the last few years many experiments have been performed that confirmed the usefulness and effectiveness of the action of copper reducing and chelating agents in antiangiogenic therapy [7,9]. Specialists in clinical research are also interested in the possibility of using zinc, which is a natural copper antagonist, in adjunctive therapy to reduce the copper level [7]. On the other hand, the results of investigations into the effect of polyphenol compounds applied in different doses on the formation and proliferation of malignant cells, using various mechanisms including the use of antioxidants to remove free radicals and copper ions chelation, as well as their effect on the cell cycle are promising enough to justify the usefulness of their application in anti-carcinogenic therapy [10-12].

### The aim of investigations

The aim of the study was to investigate the effect of dietary supplementation with zinc and polyphenol compounds, i.e. resveratrol and genistein, on the effectiveness of chemically induced mammary cancer and the changes in the content of selected elements (Zn, Cu, Mg, Fe) in tumors as compared with normal tissue of the mammary gland.

## Methods

### Animals

Fifty-six 30-days-old Spraque-Dawley female rats of 100 ± 20 g body weight, were subjected to a 10-day adaptation period. The animals were housed in stainless steel cages under controlled conditions (22 ± 1°C, a 12 h light - dark cycle), with a free access to a standard laboratory diet (Labofeed H, Poland) and drinking water. The rats were obtained from the Laboratory of Experimental Animals, Department of General and Experimental Pathology, Medical University of Warsaw (Warsaw, Poland). The animal experiments were approved by the Ethics Committee, Medical University of Warsaw.

### Experimental design

The experiment was conducted for 14 weeks (from 40 days until 20 weeks of age). After the adaptation period, the animals were divided into two experimental groups. In group 1 (DMBA+, study group n = 33), the rats were treated with a dose of 80 mg/body weight of DMBA (7,12-dimethyl-1,2- benz[a]anthracene; Sigma-Aldrich, St. Louis, MO, USA) given in rapeseed oil at 50 and 80 days of age to induce breast cancer (*adenocarcinoma*), and in group 2 (DMBA-, control group n = **23**), the rats were accommodated under the same conditions as those in Group 1; fed the same diet, but without DMBA treatment. Tumors were histopathologically evaluated to confirm their malignancy and to prove that they were *adenocarcinomas *(II and III degree). Spontaneous cancers were not found in the non-DMBA groups.

Animals from both groups were also fed diets different in bioelements. The diets were supplemented with:

- Zn - the exposed group received a daily via gavage, 0.4 mL of Zn (6.9 mg/mL; i.e. 231 mg Zn/kg diet) (as ZnSO_4_· 7H_2_O in aqueous suspension)

- Zn + resveratrol - the group received a daily via gavage, 0.4 mL of Zn (6.9 mg/mL) and resveratrol (0.1 mg/mL; i.e. 0.2 mg/kg bw) in aqueous suspension

- Zn + genistein - the group received a daily via gavage, 0.4 mL of Zn (6.9 mg/mL) and genistein (0.1 mg/mL; i.e. 0.2 mg/kg.bw) in aqueous suspension.

The animals without supplementation recived daily via gavage 0.4 mL of water.

The dose of Zn was established based on the value used in the Labofeed H diet (standard diet) (77 mg Zn/kg diet - Table 1). The polyphenols dose level was selected based on human average daily consumption (extrapolating on the rats' body weight).

**Table 1:** Declared dietary composition of Labofeed H (per kg of diet)

Protein ***(g)***		210.0	
Fat ***(g)***		39.2	
Fiber ***(g)***		43.2	
Starch ***(g)***		300.0	
Ash ***(g)***		55.0	
Vitamin A *IU*	15000	Lysine *g*	14.5
Vitamin D_3 _*IU*	1000	Methionine *g*	4.1
Vitamin E *mg*	90.0	Tryptophan *g*	3.0
Vitamin K_3 _*mg*	3.0	Threonine *g*	7.4
Vitamin B_1 _*mg*	21.0	Isoleucine *g*	17.5
Vitamin B_2 _*mg*	16.0	Valine *g*	11.0
Vitamin B_6 _*mg*	17.0	Histidine *g*	6.0
Vitamin B_12 _*μg*	80.0	Arginine *g*	13.0
Pantothenate *mg*	30.0	Phenylalanine *g*	10.0
Folic acid *mg*	5.0	Tyrosine *g*	7.8
Nicotinic acid *mg*	133.0	Choline *mg*	2750.0
Biotin *mg*	0.4		

Calcium *g*	10.0	Iron *mg*	250.0
Phosphorus total *g*	8.17	Manganese *mg*	100.0
Phosphorus saturated *g*	4.5	Zinc *mg*	76.9
Magnesium *g*	3.0	Copper *mg*	21.3
Potassium *g*	9.4	Cobalt *mg*	2.0
Sodium *g*	2.2	Iodine *mg*	1.0
Chlorine g	2.5	Selenium *mg*	0.5
Sulfur *g*	1.9		

### Samples and measurements

The animals were sacrificed by decapitation at 20 weeks of age, and tumor as well as normal tissues of the mammary gland were collected. The samples were stored at a temperature of -70°C until the test time. The mineral content was determined after wet microwave mineralization of the samples. Approximately 500-800 mg samples of tumors and normal mammary tissues or fodder were placed in a teflon vessel and with added 3.5 mL of 65% nitric acid (Suprapur^®^, Merck, Germany). **The vessels were placed **in a microwave system (MULTIWAVE, Anton Paar, Perkin Elmer) and **their content was **mineralized. The ICP-OES technique (Optima 3100XL, Perkin Elmer) was used to analyze the following elements: magnesium (Mg), iron (Fe), zinc (Zn) and calcium (Ca). Copper (Cu) content in samples was **evaluated using **an atomic absorption spectrophotometer (PU 9100) **with **air-acetylene flame. **The analysis of fodder was performed in order to check if the Zn content was in agreement with the amount declared by the producer. The obtained results were satisfactory, i.e. in agreement with the content declared by the producer (data not shown)**.

### Validation

The intralaboratory quality control of determination was **done using **the following certified Reference Materials (**percent of recovery for a given element and relative standard deviation (RSD)):**

- NCS ZC 71001 Beef Liver (**recoveries**: Cu - 118%; Fe - 109%; Ca - 115%; Zn - 105%; Mg - 96%; **RSDs - Cu - 7,2%; Fe - 4,9%; Ca - 5.0%; Zn - 3,5%; Mg - 4,6%)**

- NIST 1577b Bovine liver (**recoveries **Cu - 116%; Fe - 103%; Ca - 103%; Zn - 107%; Mg - 105%; RSDs - Cu - 13%; Fe - 1,8%; Ca - 3%; Zn - 1,8%; Mg - 1%)

Limits of detection (LOD) for: Zn - 0.03 mg/L; Ca - 1.47 mg/L; Fe - 0.17 mg/L; Mg - 0.04 mg/L and Cu - 0.13 mg/L.

### Statistics

Data are given as means ± SD (**for iron, zinc, copper and magnesium**). The results were compared to those in the control animals, in order to elucidate the possible effect of Zn or phenolic compounds supply on tumorigenesis and the mineral composition of tumor. The data were analyzed using Student's *t*-test; differences at p ≤ 0.05 were considered statistically significant. **The non-parametric *t *test (Mann - Whitney test) was applied for the parameter without normal distribution (calcium concentration)**.

## Results

### Carcinogenesis

It is a relevant issue to assess the severity of carcinogenesis expressed by tumor weight and number in particular rat groups as well as the onset time of initial tumors. Irrespectively of the diet applied, the effectiveness of DMBA-induced carcinogenesis was 100% (Table 2). Only in the group of rats supplemented with Zn + genistein, one animal developed no tumors. There were both single and multiple tumors - on average up to 6 tumors per rat, the group supplemented with Zn + resveratrol was characterized by a maximal number of 10 tumors per rat. In this group (receiving Zn + resveratrol) the first tumors appeared at 13 weeks of age, i.e. two weeks earlier than in the groups receiving Zn or Zn + genistein or only the standard diet.

**Table 2:** Cancer induction in 7,12-dimethyl-1,2- benz[a]anthracene treated groups in relation to the diet applied

diet	Tumors in group	Number of tumors in one rat	Maximum tumor weight (g)	First week at onset
1. standard diet	9/9 (100%)	1 - 5	12	15
2. zinc 6.9 mg/mL (231 mg Zn/kg diet)	9/9 (100%)	1 - 5	20	15
3. zinc 6.9 mg/mL (231 mg Zn/kg diet) and resveratrol 0.1 mg/mL (0.2 mg/kg bw)	10/10 (100%)	2 - 10	30	13
4. zinc (6.9 mg/mL) (231 mg Zn/kg diet) and genistein 0.1 mg/mL (0.2 mg/kg bw)	10/11 (91%)	1 - 6	15	15

The date refer to tumors evaluated at 20 weeks of the animals' age (decapitation time)

### Mineral content in control tissuse (DMBA-)

An appraisal was done of the effect of dietary supplementation on the content of elements in normal tissues as compared with the content of the same elements in normal mammary gland tissue in rats that were fed a standard diet only.

The applied Zn dose (exceeding threefold the amount of that element in the fodder) effectively reduced the **copper **level in normal tissue of the mammary gland in comparison with normal tissue in rats that were fed an unsupplemented standard diet. Similarly the **copper **content was reduced by over 40% for the groups that received Zn and Zn + resveratrol and by over 33% for the groups supplemented with Zn + genistein (Table 3).

**Table 3:** Altered mineral content in cancerous tissues (DMBA+) *vs *mineral content in normal tissues (DMBA-) (μg/g wet weight)

				diets					
**elements**	**DMBA**	**Standard**		**Zn**		**Zn + resveratrol**		**Zn + genistein**	
		**Mean ± SD (n)**	**RSD%**	**Mean ± SD (n)**	**RSD%**	**Mean ± SD (n)**	**RSD%**	**Mean ± SD (n)**	**RSD%**

Zn (μg/g)	-	17.6 ± 1.7 (5)	**9**.**6**	18.2 ± 1,3 (6)	**7**.**1**	18.5 ± 1.3 (6)	**7**.**3**	17.2 ± 2.3 (6)	**13**
	+	17.6 ± 0.9 (7)	**5**.**1**	17.3 ± 1.3 (8)	**7**.**5**	19.2 ± 1.2 (8)	**6**.**1**	17.0 ± 1.1 (10)	**6**.**6**
Cu (μg/g)	-	2.57 ± 0,79 (5)	**30**	1.36 ± 0.22 (6)**	**15**	1.47 ± 0.19 (6)**	**12**	1.74 ± 0.45 (6)	**26**
	+	2.92 ± 0.85 (7)	**29**	2.63 ± 0.52 (8) *	**19**	3.45 ± 0.82 (8) *	**23**	2.87 ± 0.79 (8) *	**27**
Fe (μg/g)	-	17.7 ± 3.1 (5)	**17**	18.6 ± 2.2 (6)	**12**	17.1 ± 2.4 (6)	**13**	16.3 ± 2.1 (5)	**12**
	+	50.7 ± 22.6 (7) *	**44**	28.9 ± 6.3 (8) *	**21**	51.4 ± 19 (8) *	**36**	33.4 ± 9.6 (10) *	**28**
Mg (μg/g)	-	224.4 ± 33.8 (5)	**15**	263.5 ± 7.66 (6)	**2**.**9**	228 ± 14.7 (6)	**6**.**4**	236.8 ± 18.7 (6)	**7**.**8**
	+	153.6 ± 10,5 (7) *	**6**.**8**	166.5 ± 11.3 (8) *	**6**.**7**	146.4 ± 12.1 (8) *	**8**	160.8 ± 9.3 (10) *	**5**.**8**

*differences (p ≤ 0.05) between concentrations of metals in DMBA (+) and DMBA (-) groups of each type of diet

**differences (p ≤ 0.05) between concentrations of metals in each type of diet (DMBA-) relative to standard diet (DMBA-)

SD - standard deviation; RSD - relative standard deviation (%); n- number of samples

**The Ca content in normal tissue was significantly elevated in the group supplemented with Zn + genistein in comparison with normal tissue in rats that were fed an unsupplemented standard diet **(Table 4). No differences were found with respect to the remaining elements (Table 3).

**Table 4:** Altered calcium content in cancerous tissues (DMBA+) *vs *calcium content in normal tissues (DMBA-) (μg/g wet weight)

Diet	DMBA(-) Mean (confidence interval) (n)	DMBA(+) Mean (confidence interval) (n)	p
**Standard**	**234.8** **(73.0 - 754.8)** **(5)**	**404.4** **(178.0 - 916.7)** **(7)**	**< 0.343**
**Zn**	**216.4** **(54.8 - 854.2)** **(6)**	**97.8** **(82.4 - 115.9)** **(8)**	**< 0.949**
**Zn + resveratrol**	**83.6** **(58.5 - 119.5)** **(6)**	510.1* (195.4 - 1334.7) **(8)**	**< 0.001**
**Zn + genistein**	64.7** (60.9 - 68.8) **(5)**	219.7* (97.9 - 493.6) **(10)**	**< 0.001**

*differences between concentrations of calcium in DMBA (+) and DMBA (-) groups of each type of diet

**differences between concentrations of calcium in each type of diet (DMBA-) relative to standard diet (DMBA-)

The mean values and confidence intervals were calculated for data transformed in logarithms, and then retransformed after calculations. *P *values were calculated using non-parametric test (Mann-Whitney test); n - number of samples.

### Zinc and calcium content in tumors

The applied excessive zinc supply did not affect the amount of that element in the malignant tissue, irrespectively of whether zinc was supplied alone or in combination with polyphenols in doses corresponding to mean daily human intake calculated per rat's body weight (Table 3).

The Ca content in tumors was significantly elevated **in the groups supplemented with Zn + resveratrol and Zn + genistein. Generally, considerable differences occurred in Ca content, irrespectively of the group or diet **(Table 4).

### Copper, iron and magnesium content in tumors

The applied doses of zinc, resveratrol and genistein were found to significantly change the **copper, iron **and **magnesium **content in malignant mammary gland tumors as compared with normal tissue (Table 3).

The **copper **content increased by as much as 135% in the group supplemented with Zn + resveratrol, by 93% in the group supplemented with Zn, and by **64**% in the group supplemented with Zn + genistein. No significant differences were found in the group of rats that were fed a standard diet only (Table 3).

The **iron **content in tumors also significantly increased: by **more than **200% in groups of rats fed a standard diet and **those **supplemented with Zn + resveratrol; by 100% in the groups supplemented with Zn + genistein, and by 55% in tumors of the rats that received a diet supplemented with Zn (Table 3).

The **magnesium **content in malignant tissue as compared with normal tissue was significantly reduced by over 30% in all groups (Table 3).

## Discussion

In our *in vivo *investigations the applied dose of zinc was three times higher than the amount of zinc normally found in the fodder (also in combination with polyphenols) and this effectively reduced the **copper **content in normal tissue of mammary gland in the rats of the control group as compared with the rats fed a standard diet only. Therefore it can be supposed that the applied dose of zinc and polyphenols had a positive effect as it reduced the amount of copper in the body without causing any toxic effect such as loss of appetite (unwillingness to eat) or loss of body weight.

However, DMBA-induced chemical carcinogenesis changed the situation completely. Irrespectively of the applied diet, no decrease of **copper **content was observed in already existing tumors. On the contrary, the supplementation with zinc or zinc + polyphenols resulted in strong **copper **accumulation in malignant tumors. Thus it seems that a key role in this effective accumulation of copper was played by a combination of Zn and DMBA, and to some extent by polyphenols.

Copper plays a significant role in the process of neoplastic angiogenesis. Malignant tumors can develop relatively easily up to the size of 1-2 mm^3^. Their further growth requires the formation within the tumor of a network of blood vessels that ensure better cell nourishment, and also allow their expansion in the form of metastases [7]. The process of angiogenesis begins as a result of metabolic oxidative stress in tumor cells. The first stage of this process always involves the activation of endothelial cells. The **copper **ions have a stimulating effect on the proliferation process, through their activating role with respect to various growth factors such as VEGF (vascular endothelial growth factor), TNF (tumor necrosis factor), EGF (epidermal growth factor) or IL-1 (interleukin 1). The factors that have been activated bind with receptors in endothelial cells. As a result, the cell passes from phase G_0 _to phase G_1 _and the cell proliferation process is activated. Besides, the presence of copper is required for some proteins to obtain antigenic properties, e.g. for ceruloplasmin, angiogenin or glycyl-L-histydyl-L-lysine tripeptide [7]. The investigations of Brem and Wotoczko-Obadio [13] showed that after decreasing copper concentration by using penicillamin and a special diet poor in copper the proliferating cell can enter phase G_0 _again, or apoptosis can occur, as a result of which the angiogenic activity of VEGF, TNF, EGF or IL-1 is inhibited [13].

Therefore, because of a very important role of copper in tumor angiogenesis, it seems necessary to search for compounds that would have a chelating effect or that would reduce its amount in the bloodstream. Zinc is a natural copper antagonist. Zinc-induced metallothioneins in intestinal lumen bind to copper thus inhibiting its absorption into the bloodstream. Reduction of copper concentration to 20% of its physiological level is considered to be a favorable therapeutic response **(various diseases of fibrosis and/or inflammation) **[14-16]. It seems that this low copper concentration is enough to activate Cu-dependent enzymes and at the same time to inhibit tumor growth. **In order to decrease the copper content more rapidly, zinc ions are used as adjunct medication together with other copper chelates (d-penicillamine, thiomolybdate or biogenic methanobactin) **[12,16,17]. It is known that long-term increased zinc supplementation has no toxic results, which can be seen in the treatment of Wilson's disease [17,18]. One drawback of the antiangiogenic zinc treatment is that it is only effective when supplied over long periods of time. In order to reduce the Cu level to 20% of its physiological value zinc must be supplied in a dose three times as high as the average daily demand for zinc, i.e. in pharmacological doses of 40-50 mg/day, and for a period of up to six months [18].

Zinc is a chemical element that takes part in proliferation and reproduction of cells, in transcription and repair of DNA damage as well as in storage and liberation of hormones. It also plays an immunological role and provides an important antioxidant defense against free radicals (by way of copper and zinc superoxide dismutation, antagonistic activity with respect to transition metals, protection against the oxidation of sulfhydryl groups of enzymes as exemplified by δ-aminolevulin desaturase, induction of metallothionein expression as well as regulation of apoptosis). It can be said that intracellular **zinc **concentration is decisive for the future of the cell, its proliferation, differentiation, and cell death by necrosis. Many investigations confirmed the phenomenon of zinc accumulation in human mammary gland tumors as well as in chemically induced tumors in animals by using N-methyl-N-nitrosourea (MNU) [19,20]. **Zinc is essential for cell proliferation and differentiation, especially for the regulation of DNA synthesis and mitosis (as it is generally known that neoplastic cells undergo uncontrolled proliferation)**.

However, in our investigations in which the rats with DMBA-induced tumors were supplemented with zinc every day for 100 days, no differences in **zinc **content were found in comparison with normal tissue, irrespectively of the diet applied. Even the comparison of control groups supplemented with zinc with control groups fed a standard diet showed no differences in zinc content. It seems that also in this case the neoplastic tissue did not accumulate more zinc (in spite of increased proliferation), unlike in the case of iron and copper. It was shown in many studies that zinc insufficiency in the body may lead to the development of different forms of cancer, whereas zinc supplementation was proven to inhibit cancer development [21,22]. So it might seem that zinc supplementation applied in this study should, at least by competing with copper and iron, effectively weaken the process of cell proliferation. However, this was not the case and so it can be supposed that cancer cells have a specific strong ability to accumulate first of all iron, but also copper, i.e. the elements that are indispensable for triggering oxidative stress.

Iron not only facilitates the appearance of oxidative stress, but also activates proinflammatory cytokines and in the case of their excess leads to **intracellular **hypoxia [5,23]. From epidemiological studies it results that the risk of cancer is considerably increased in patients with a large iron supply in comparison with those with normal or below normal content of this element [23]. It was shown that breast cancer cells have 5-10-fold more receptors for transferrins (TfR1) than normal cells of the mammary gland, probably because they receive a higher amount of iron. Meanwhile, another type of transferrin receptors known as TfR2 was also found. These receptors are present in many malignant cells and may effectively contribute to the iron's increased ability of absorption [23]. The increased iron supply in the diet increases the frequency of occurrence of carcinogen-induced mammary cancer in rats and estrogen-induced kidney cancer in hamsters [23]. Thus it is very probable that by affecting iron metabolism and disturbing the process of its accumulation in malignant tissues [23] it may be possible to prevent a number of different cancers**. Some researchers hold that the lowering of iron content may be a way to treat cancer, because in this way the proliferation of tumors is limited (investigations performed on rats)**. In the investigations performed both *in vitro *and *in vivo *it was shown that deferoxamine, an iron-chelating agent that decreases its bioavailability is effective in tumor cells treatment [23]. The results obtained by us concerning the increased iron concentration in tumors are in agreement with the results of other authors [19,24].

The present investigation showed that, irrespectively of the type of supplementation applied, there was a considerable increase of **iron **content in tumors in comparison with normal tissue, also for the standard group. This may mean that supplementation with zinc and polyphenols had no effect on the process of iron absorption by malignant tissues.

Similarly, there was no stimulating effect of the diet applied on the magnesium content in malignant tissue. In this case it seems that the carcinogen while causing mutation and tumor formation, very strongly controls the demand of those tissues for the given element. Irrespectively of the applied diet there was a decrease of **magnesium **in malignant tissue in comparison with **its **content in normal tissue of healthy rats that received an analogical diet **(i.e. the same diet in the control group (DMBA-) and the examined group (DMBA+)**. **This effect is most pronounced in the groups supplemented with Zn and Zn + resveratrol**. The reduced **magnesium **content was also found in patients with nasal polyp, papilloma or laryngeal carcinoma [25]. The effect of magnesium on the development of neoplasm may be due to oxidative stress, DNA oxidation and the resulting mutagenesis, as well as by affecting the DNA repair mechanisms that are critical for the maintenance of genome stability [2].

In the present investigation resveratrol and genistein were used as they are both strong antioxidants and chemopreventive compounds [10,11]. The hydroxyl groups of polyphenols react with free radicals and form more stable fenoxyl complexes. Besides, polyphenols while causing complexation of Cu (II) and Fe (II) ions, may inhibit the activity of some enzymes, e.g. of xanthine oxidase and processes increasing the free radicals formation [26,27]. The chemopreventive activity of polyphenols is multifactorial - ranging from inhibition of cytochrome P-450 isoenzymes, induction of phase II enzymes (UDP-glucuronyltransferase and NADPH oxydoreductase (28), inhibition of NFκBI and AP-1 (activator protein 1) as well as of DNA topoisomerases I and II and helicases, inhibition of angiogenesis, inhibition of cyclooxygenase 1/2 enzymes, inducing NO synthesis and increasing the p-53 protein expression), and finally to exhibiting similar characteristics to steroid sex hormones [12,28-30]. However, in investigations with the use of various cell lines and animal models resveratrol revealed opposing activities: estrogenic activity or antiestrogenic activity [29]. In the estrogen-dependent cell line of mammary cancer, in the case of estrogen deficiency resveratrol behaved like a competitive estrogen receptor agonist and stimulated the cell proliferation [29,31], whereas in the presence of estrogen it acted as an estrogen detector antagonist.

Similarly, genistein exhibits antiproliferative activity with respect to human cells of breast cancer MCF-7 when applied in large pharmacological doses (> 10 μM), and estrogenic activity when applied in small physiological doses (0.1-1 μM) which however is considerably weaker than that of natural estrogen [32,33]. In the majority of investigations performed on animals genistein was found to inhibit proliferation of mammary tumors as well as of other forms of cancer [34].

One of the criteria showing that the animals are prone to tumor induction is the moment when malignant calluses are felt by palpation. In the case of our investigations it was found that in the rats that were fed a standard diet the first tumors appeared at the same time (15th week) as in the groups that were supplemented with Zn and/or Zn + genistein. In the group of rats that received Zn + resveratrol the first tumors appeared two weeks earlier than in the remaining groups (which corresponds to a period of about 10-12 months in humans) [35]. What is the reason of the difference between the rate of tumorigenesis in the rats that were fed a standard diet and those supplemented with Zn or Zn + genistein, and an increased rate of tumor formation in the group of rats whose diet was supplemented with Zn ions + resveratrol? In the studies of Win et al. [36] on the effect of genistein and resveratrol on induction of plasmid DNA impairments by reactive oxygen species (ROS) that formed in the medium H_2_O_2_/Cu(II) and hydroquinone/Cu (II) it was shown that the activity of these two polyphenols proceeds via different mechanisms. The presence of genistein in micromolar concentrations considerably inhibits DNA damage, however not by affecting the Cu (II)/Cu (I) redox cycle or in reaction with H_2_O_2_, but by working as an effective agent that has the capacity to sweep away the reactive oxygen species (ROS) that have formed. Contrary to that, resveratrol present in similar concentrations (25 μM-200 μM) increases DNA breakage induced by H_2_O_2_/Cu (II). Many researchers showed that it is resveratrol (and not genistein) that increases DNA damage in the presence of Cu (II) ions. Neoplastic cells accumulate great amounts of copper, including that bound in the nucleus. Thus the formed complex resveratrol/Cu (II) after binding to DNA may induce DNA breakage [36]. **On the other hand**, many *in vivo *investigations confirmed that resveratrol supplemented in the diet may reduce the occurrence and multiplicity of the chemically induced mammary tumors, first of all by affecting COX 2, expression of metalloproteinases, and the nuclear factor κB [37]. As a result the maturation of the mammary gland is enhanced, which in turn leads to reduction of cell proliferation and an increase of apoptosis in the epithelium of mammary cells [37,38]. However, there are also reports stating that short-term resveratrol supplementation (depending on the dose) before pubescence may result in endocrine disturbances and cause irregular cycles with prolonged oestrus phase, which in turn increases the multiplicity of mammary tumors in rats [39]. Estrogens are known as important natural mitogenic factors in human breast, which is connected with an increased breast cancer risk. Resveratrol exhibits anticarcinogenic activity on animal models [39] in the case of skin cancer, when administered to adult animals. It is worth noting that during the perinatal period the organism is very sensitive and so the high level of exposure to estrogens during pregnancy increases the risk of mammary cancer in female offsprings [39]. However, available data as concerns the interaction between mammary cancer and estrogen level are still inconsistent. The timing and level of exposure to estrogenic chemicals are apparently important. There are reports claiming that genistein administered to rats in the perinatal, neonatal and pre-pubescence periods also induces irregular oestrus cycles related to prolongation of the oestrus phase [39,40] and thus may accelerate the development of mammary carcinoma. In another paper [41] it was shown that phytoestrogens, including genistein, when supplied during prepubescence reduce the risk of mammary cancer development in the future.

As concerns resveratrol, there are few investigations concerning this period. In adult rats resveratrol when administered by a stomach tube or with the diet, significantly inhibited mammary oncogenesis induced by MNU or DMBA [42,43]. On the other hand, in the paper of Sato et al. [39] resveratrol in a dose of 100 mg/kg body weight accelerated the appearance of MNU-induced mammary carcinomas, but no such activity was observed when resveratrol was given in a dose of 10 mg/kg body weight.

Cancer incidence and multiplicity were significantly higher in the high-dose group, whereas latency was unchanged. As the majority (80%) of MNU-induced mammary carcinomas are hormone-dependent resveratrol appears to increase the ER - and/or PgR-positive cells presumed to be the progenitors of hormone-dependent carcinomas resulting in a higher mammary carcinoma yield [44,45].

In our investigations the lack of anticarcinogenic activity of dietary supplementation with zinc ions and polyphenols, or even acceleration of mammary oncogenesis in animals on the zinc + resveratrol supplemented diet, may be related to sexual immaturity of the rats.

## Conclusions

Summing up, it should be emphasized that the increased supply of zinc alone or in combination with either resveratrol or genistein resulted in a decrease of copper content in normal tissue of the mammary gland, whereas in the case of malignant tissue an opposite effect was observed. On the basis of quantitative analysis of selected elements we found - irrespectively of the diet applied - great accumulation of copper and iron, which are strongly prooxidative, with a simultaneous considerable decrease of the magnesium content in DMBA-induced mammary tumors. The combination of zinc supplementation with resveratrol resulted in particularly large differences in the amount of the investigated elements in tumors as compared with their content in normal tissue. In addition, the supplementation with zinc + resveratrol significantly increased the rate of appearance of the first tumors that could be felt by palpation as well as their multiplicity, which is indicative of accelerated oncogenesis. Perhaps a given phytoestrogen can have opposing effects on mammary cancer risk, depending on the time of exposure. The exact mechanism is not known, especially as each of the examined compounds is rather considered to be an ally in the fight against cancer. However, the presented results make us wonder if the diet supplementation with several antioxidants at the same time is likely to afford the expected result of inhibiting the risk of carcinogenesis, especially in children and young people before pubescence.

## Abbreviations

AP-1: Activator protein 1; Ca: Calcium; COX 2: Cyclooxygenase-2; Cu: Copper; DMBA: 7,12-dimethylbenz[a]anthracene; EGF: Epidermal growth factor; ER: Estrogen receptor; Fe: Iron; ICP OES: Inductively coupled plasma optical emission spectrometry; IL-1: Interleukin 1; i.e, that is; Mg: Magnesium; MCF-7: Human breast adenocarcinoma cell line; MNU: N-methyl-N-nitrosourea; NFκBI: Nuclear factor kappa B; PgR: Progesterone receptor; ROS: reactive oxygen species; RSD: Relative standard deviation; TM: Tetrathiomolybdate; TNF: tumor necrosis factor; VEGF: Vascular endothelial growth factor; Zn: Zinc.

## Competing interests

The authors declare that they have no competing interests.

## Authors' contributions

BB planned, designed and carried out the experiment. DS planned, designed and carried out the experiment, AT coordinated the study. All authors read and approved the final manuscript.
